# Boto (*Inia geoffrensis*—Cetacea: Iniidae) aggregations in two provisioning sites in the lower Negro River—Amazonas, Brazil: are they related?

**DOI:** 10.7717/peerj.6692

**Published:** 2019-04-17

**Authors:** Waleska Gravena, Tomas Hrbek, Vera Maria Ferreira da Silva, Izeni Pires Farias

**Affiliations:** 1 Instituto de Saúde e Biotecnologia (ISB), Universidade Federal do Amazonas (UFAM), Coari, Amazonas, Brazil; 2 Laboratório de Evolução e Genética Animal (LEGAL), Universidade Federal do Amazonas (UFAM), Manaus, Amazonas, Brazil; 3 Laboratório de Mamíferos Aquáticos (LMA), Instituto Nacional de Pesquisas da Amazônia (INPA), Manaus, Amazonas, Brazil

**Keywords:** River dolphins, Microsatellite markers, Sex markers, Human interactions

## Abstract

The Negro River currently has seven floating houses where tourists can feed and interact with botos, each with its own history of how these aggregations were formed. Some keepers say these groups are familial, even reporting individuals being born into the group. However, behavioral studies have shown that botos are solitary, only forming groups at feeding areas and during the mating season. In the present study we used 12 microsatellite and molecular sex markers to characterize relationships within and between two boto aggregations (ten and seven botos each) in the lower Negro River. Molecular sexing revealed that all botos sampled from both aggregations were males. This may be explained by habitat preference, as male botos are primarily found in the main channels of large rivers, whereas females prefer more protected areas, such as flooded forests and its channels and lakes. Most of the animals were unrelated within each aggregation, demonstrating that these aggregations are not normally formed due to kinship bonds, but are exclusively for feeding, as botos learn that these places provide easy access to food. This study provides important information that helps us understand how human interaction is affecting the social structure and behavior of these animals.

## Introduction

Alliances and aggregations are formed by many species of mammals for many different reasons. African elephants (*Loxodonta africana*) move long distances as a group for safety purposes (Thouless, 1995). Female and juvenile coatis (*Nasua narica*) form groups to gain access to limited food resources (Gompper, 1997; Gompper, Gittleman & Wayne, 1998). Southern flying squirrels (*Glaucomys volans*) form groups during the cold season to gain thermoregulatory benefits (Layne & Raymond, 1994). In a number of primate species, males typically form alliances to gain access to reproductive opportunities or to increase their competitive advantage in territorial disputes (Pusey & Packer, 1987; Kappeler & Van Schaik, 2002). Some mammalian females form alliances or social bonds mainly to compete for food resources while nursing, as observed in lions *Panthera leo* (Heinsohn & Packer, 1995), or for protection of their offspring, such as seen in baboons *Papio cynocephalus ursinus* (Silk et al., 2009) and feral horses *Equus ferus caballus* (Cameron, Setsaas & Linklater, 2009).

Many odontocetes form groups that regularly swim together as a unit (Norris & Dohl, 1980). Groups are formed for a variety of purposes, including feeding, breeding, communication, social interaction, learning and defense (Norris & Dohl, 1980). The formation of groups for these reasons, and as basis for alliances, are very common in the family Delphinidae, as is observed in *Tursiops* sp., *Lagenorhynchus acutus*, *Sotalia guianensis*, among others (Parsons et al., 2003; Connor et al., 2011; Mirimin et al., 2011;  Lunardi & Ferreira, 2014). However, it is not common for river dolphins of the families Platanistidae, Lipotidae and Iniidae to form stable groups (Braulik, 2014; da Silva & Martin, 2014; Smith, 2014). The South Asian River dolphin, or susu (*Platanista gangetica*) is generally observed alone or in loose groups of two or three individuals, but may occasionally be observed in aggregations of 20 individuals or more (Braulik, 2014). Very little is known about the social organization of the Yangtze River dolphin, or baiji (*Lipotes vexillifer*). Group sizes were generally of three or four individuals, with as many as 16 observed in a single location, but it is unknown if these groups were based on stable social affiliations or were ephemeral associations related only to common use of resources (Smith, 2014).

The Amazon River dolphins (*Inia geoffrensis*), also known as botos, are solitary animals and are rarely seen in groups of more than four individuals; even when found in groups, these groups are not considered to be of stable long-term relationships (Martin & da Silva, 2004). The only stable bonds observed in botos are between a mother and her calf, during the lactation period (Best & da Silva, 1989a). Aggregations of up to several dozen botos often occur at waterway confluences and lake system entrances, but these are ephemeral and based on exploitation of a food resource (da Silva & Martin, 2014). Botos, as other river dolphins, including the susu, usually have fixed home ranges where they may hunt individually as well as cooperatively (Herman, 1980; Martin & da Silva, 2004; da Silva & Martin, 2014).

Cetaceans use a variety of strategies to find and capture their prey (Heithaus & Dill, 2009). While most species of dolphins are highly social, with groups covering large areas in search for food, other species appear to have fixed home ranges and can be seen in the same area day after day (Norris & Dohl, 1980). The latter behavior is common in river dolphins, including the susu, and the boto (Herman, 1980; Martin & da Silva, 2004; da Silva & Martin, 2014). Both species exhibit a wide range of prey capture methods and hunt individually as well as cooperatively in groups (Ballance, 2009).

The boto is the largest freshwater cetacean and is widely distributed throughout the Orinoco, Amazon River basins (Best & da Silva, 1989b) and Araguaia-Tocantins River basins (Hrbek et al., 2014). Despite occurring over an enormous area (da Silva & Martin, 2014), the botos’ home range is still poorly known, which is one of the priorities of its action plan (da Silva & Martin, 2010). Site fidelity was studied (McGuire & Henningsen, 2007); however, results were limited due to behavioral, morphological, and ecological characteristics. Daily movements of up to 20 km are typical, and individuals move at sustained swimming speeds of 3–6 km/h (da Silva & Martin, 2014).

**Figure 1:**
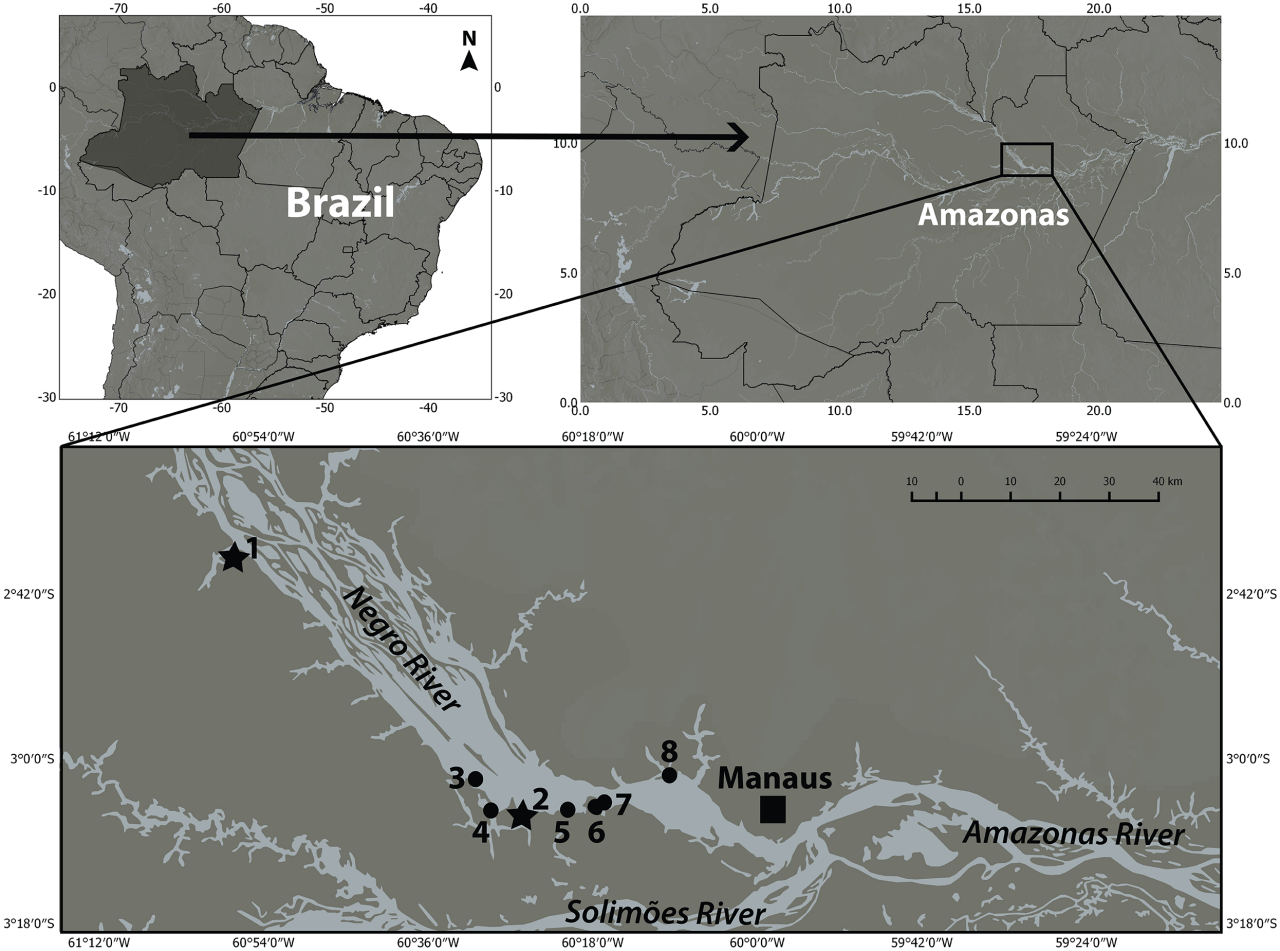
There are currently seven places where botos are provisioned in the lower Negro River. However, when samplings were carried out in 2005, there were only Flutuante AMA BOTO in Novo Airão (1) and Flutuante do Boto in the Ariaú River (2), which are marked with stars. The other places, marked with a circle, Flutuante Recanto do Boto in the Acajatuba River (3), Flutuante do Davi in the Negro River (4), Flutuante do Samuel in the Negro River (5), Flutuante do Jacaré in the Negro River (6), Flutuante da Cachoeira do Castanho also in the Negro River (7), and Flutuante do Cristovão in the Tarumã-Mirim River (8) (no longer operating). Figure adapted from Alves et al. (2011).

In the state of Amazonas—Brazil, in the lower Negro River near Manaus, there are currently seven places where botos are provisioned and tourists are allowed to swim with them. These activities, here entitled boto aggregations, occur around wooden floating houses that have a submerged platform where tourists, along with keepers of the establishments interact with the animals (Alves et al., 2013). In general, aggregations are on average made up of eight individuals per site (VMFS, pers. obs., 2018). Each individual is identifiable by scars, pigmentation patterns and other marks, with each animal receiving a name from the keeper. Interactions between botos and tourists occur daily and with no pre-established time. The keepers feed the animals every day even when no tourist interactions occur, but as soon as a group of tourists arrives at the floating house larger quantities of food are offered. During the interactions, not all animals are fed at the same time and some do not get any food at all. This has been demonstrated in the aggregation at Novo Airão (Barezani, 2005;  Romagnoli, 2010), but has also been observed in all other aggregations. Therefore there is no apparent feeding pattern, some animals come to feed every day for days at a time, while others come sporadically and disappear for days or even months (Barezani, 2005; Angélica Nunes, pers. com., 2015) while new animals appear at the aggregation and begin to interact with tourists (VMFS, pers. com., 2018). One of the provisioning sites that has been running continuously since 1998 is in Novo Airão (number 1 on Fig. 1) (Barezani, 2005;  Romagnoli, 2010; Alves et al., 2011). In 2004 another aggregation was formed at the mouth of the Ariaú River, at the Ariaú Amazon Towers Hotel in Iranduba county (number 2 on Fig. 1) ( Romagnoli, 2010). Six other aggregations are shown in Fig. 1. In the Novo Airão aggregation, the keepers believe that the botos belong to a family group containing males and females, as well as offspring born within the group, but there are no data to confirm this supposition. Considering the importance of kinship in the formation of social bonds and taking into consideration that boto populations of the Negro River have not been genetically characterized, the goals of this study were to (1) genetically characterize individuals in the Novo Airão and Ariaú aggregations, (2) estimate genetic diversity within and between these aggregations, and, (3) determine genetic relationships between individuals (e.g., sibling, parent–offspring).

## Materials and Methods

The two floating houses we sampled were Flutuante Boto-cor-de-rosa, currently known as AMA BOTO in Novo Airão (180 km north west of Manaus) in October 2004 and July and December 2005, and Flutuante do Boto, located in the mouth of the Ariaú River in Iranduba County (60 km northwest of Manaus) in March 2006. The two sites are 90 km away from each other (Fig. 1) and were the only ones in operation at the time samples were collected . Collecting permits were provided to IPF by IBAMA/ICMBIO (No. 11325-1 and No. 13462-1). The protocol for handling and removing tissue samples from live animals was approved by the National Research Institute of the Amazon (INPA) (No. 001/2013).

We led three different sampling expeditions in Novo Airão and one expedition in Ariaú. Collection of all samples followed the same methodology. For two consecutive days, we collected samples from all the animals that visited the aggregation, totaling around six hours of interaction with the animals per day. A total of 17 samples were analyzed—ten from botos of Novo Airão and seven from botos from the Ariaú River.

Epithelial tissue from the botos was collected using a modified thimble (Cunha, Azevedo & Lailson-Brito Jr, 2010) designed specifically to collect dolphin skin samples, and adapted by us for use on animals that allow human contact. We scraped the thimble along the pectoral fluke, removing long and thin superficial threads of skin. The animals demonstrated no visible reaction to sampling, and we observed no scarring later on. All samples were stored in 95% ethanol and deposited in the official animal tissue collection of the Amazonas Federal University (Coleção de Tecidos de Genética Animal—CTGA/UFAM).

We used skin fragments of approximately 3 mm for DNA extraction. Total DNA was isolated using the Genomic Prep Cells and Tissue DNA Isolation Kit (GE Healthcare, Chicago, IL, USA). The extraction was assessed both qualitatively and quantitatively through electrophoresis in 1% agarose gel.

Twelve microsatellite primers were used for the kinship analysis, five of which had been developed for *Tursiops truncatus*, Ttr11, Ttr48, Ttr63, TtrRC12 (Rosel, Forgetta & Dewar, 2005), TtruAAT40 (Caldwell, Gaines & Hughes, 2002) and one which was developed for *Tursiops aduncus*, MK9 (Krützen et al., 2001). These primers have proven to be informative for *Inia geoffrensis* (Gravena et al., 2009). The other six primers (Ig3A1, Ig11B1, Ig2G1, Ig1F1, Ig2B1, Ig1B1) were developed for the focal species (Gravena et al., 2009). PCR conditions follow those described in Gravena et al. (2009). All primers were labeled with a fluorescent dye following Schuelke (2000). Amplified microsatellite fragments were analysed in the MegaBACE™ 1000 automatic sequencer (GE Healthcare). We inferred the size of the amplified fragments using the ET 400 ROX size standard (Amersham BioSciences, Piscataway, NJ, USA) in the Genetic Profiler and Fragment Profiler programs (GE Healthcare).

Sex of all individuals was determined via PCR using one pair of primers for the ZFX gene and one pair for the SRY gene found on the X and Y chromosomes, respectively (Rosel, 2003). Although the primers were initially developed for *Tursiops truncatus*, they have been tested for other species of cetaceans, including botos (Rosel, 2003). PCRs were carried out simultaneously with both primer pairs using standard PCR conditions (Rosel, 2003). We used a minimum of two PCR reactions for each sample in order to assess reliability of the results. In each PCR we also ran two control animals of known sex, a male and a female, and a negative control. PCR results were observed on 2.5% agarose gel and compared with a 100 bp DNA size standard. An individual was considered male if two different bands were observed in the gel, one representing the ZFX gene amplicon (382 bp) and one the SRY gene amplicon (339 bp), whereas females had only one band representing the ZFX gene amplicon.

The microsatellites were analyzed for the presence of null alleles, allelic dropout and stutter using the program MICRO-CHECKER v2.2 (Van Oosterhout et al., 2004). Matrix of genotypes is available at https://github.com/legalLab/datasets. Deviation tests of Hardy–Weinberg equilibrium proportions and linkage disequilibrium were performed in ARLEQUIN v3.5 (Excoffier & Lischer, 2010). Genetic variability was assessed in terms of the number of alleles per locus, heterozygosity (observed and expected) and the estimated gene diversity within each aggregation. Considering that some of these estimates suffer influence of sample size (Leberg, 2002), we implemented a rarefaction analysis and calculated allelic richness (AR) and private allelic richness (PAR) in the program HP-Rare (Kalinowski, 2005), so that the number of alleles and allele richness estimates could be compared between localities. To show the power of the group of chosen markers we also estimated the joint probability of genetic identity (*IC*) according to Paetkau et al. (1995) and the joint probability of paternity exclusion at all loci (*QC*) using Weir’s (1996) method.

To infer the most likely number of biological groups existing within our sample, we used the program STRUCTURE 2.3.4 (Pritchard, Stephens & Donnelly, 2000) to generate posterior probabilities for different numbers of groups using the ‘admixture’ and ‘correlated-allelic-frequencies’ models. We explored the possibility of our sample containing from one to three biological groups. Assignment space was explored with 1,000,000 MCMC chains, preceded by 100,000 MCMC chains discarded as burn-in. Each analysis was repeated 20 times from a different randomly selected starting point. The most likely number of biological groups (K) was inferred using Bayes’ Rule (Suchard, Weiss & Sinsheimer, 2001; Pericchi, 2005).

Analysis of molecular variance (AMOVA) (Excoffier, Smouse & Quattro, 1992) was employed to describe the amount of differentiation and gene flow (Neigel, 2002) between the two aggregations. We analyzed the data using Wright’s *F*_*ST*_ (Wright, 1969) rather than Slatkin’s *R*_*ST*_ (Slatkin, 1995), as the variance of the latter tends to be greater when less than 20 loci are analyzed (Gaggiotti et al., 1999).

Estimates of recent migration rates between the Novo Airão and Ariaú aggregations were calculated using the program BayessAss v3.0 (Wilson & Rannala, 2003). Analyses were carried out assuming two geographic groups. Each MCMC chain had 5 ×10^6^ steps, with 10^4^ discarded as burn-in during posterior analyses.

Additionally, the inbreeding coefficients (*F*_*IS*_) (Weir & Cockerham, 1984) using ARLEQUIN v3.5 (Excoffier & Lischer, 2010) were obtained. Analyses were carried out assuming two geographic groups. *F*_*IS*_ represents the average probability that any particular individual has two copies of an allele that is identical-by-descent at any particular locus, i.e., the average probability of expected homozygosity within each group.

We estimated the levels of relationship between pairs of individuals from the two aggregations using the relatedness coefficient (*r*) of Lynch and Ritland rLR99 (Lynch & Ritland, 1999) and Queller and Goodnight rQG (Queller & Goodnight, 1989) using the programs KINGROUP (Konovalov, Manning & Henshaw, 2004) and IDENTIX (Belkhir, Castric & Bonhomme, 2002). Assuming that the study group is not inbred (sensu pedigree inbreeding), *r* values of 0.12, 0.25, 0.50 or greater than 0.50, were considered as third order relatives (cousins, CO), second-order relatives (half siblings, HS) and first-order relatives (full siblings, FS, or parents and offspring, PO), respectively. Both positive and negative values near zero are representative of unrelated individuals.

## Results

### Genetic diversity and population structure

We detected no null alleles, allelic dropout or genotyping errors in any of the 12 loci of the genotyping panel. No significant linkage disequilibrium was detected between pairs of loci from either of the two locations, and no deviations from the Hardy–Weinberg proportions were detected in any of the loci. Despite having 5.88% of missing data, the probabilities of genetic identity (*IC* = 1.8429 × 10^−8^) and paternity exclusion (*QC* = 0.99629220) indicated that theses markers provide a robust set of tools for analyses of social structure.

Genetic statistics per locus and per sampling locality are shown in Table 1. Observed and expected heterozygosity ranged from 0.10 to 0.90 and from 0.19 to 0.84, respectively. Genetic variability parameters were quite homogeneous between the two different localities (Table 2). The mean expected heterozygosity in both groups was 0.58.

**Table 1:** Characteristics of the 12 microsatellite loci analyzed for *Inia geoffrensis* aggregations from Ariaú and Novo Airão considering, separately, the groups of individuals by collection site.

	Ig3A1	Ig11B1	Ig2G1	Ig1F1	Ig2B1	Ig1B1	MK9	Ttr11	Ttr48	Ttr63	TtrRC12	TtruAAT40
Ariaú												
Allele	2	5	2	5	3	2	3	6	2	4	3	2
*Ho*	0.50	0.71	0.43	0.85	0.71	0.60	0.50	0.71	0.57	0.71	0.57	0.42
*He*	0.57	0.72	0.36	0.83	0.62	0.56	0.70	0.84	0.44	0.57	0.69	0.36
*p*	1.00	0.89	1.00	0.36	1.00	1.00	0.12	0.18	1.00	1.00	0.17	1.00
NovoAirão												
Allele	3	7	2	5	4	2	4	5	3	5	2	5
*Ho*	0.22	0.80	0.10	0.90	0.50	0.50	0.80	0.89	0.60	0.40	0.70	0.30
*He*	0.48	0.74	0.19	0.58	0.68	0.40	0.66	0.77	0.68	0.74	0.52	0.63
*p*	0.11	0.60	1.00	0.44	0.81	1.00	0.28	0.88	0.78	0.08	0.52	0.009
All												
Allele	3	8	2	6	5	2	4	7	3	6	3	5
*Ho*	0.30	0.76	0.23	0.88	0.58	0.54	0.68	0.81	0.58	0.52	0.64	0.35
*He*	0.55	0.73	0.27	0.79	0.65	0.48	0.65	0.77	0.62	0.70	0.55	0.52
*p*	0.16	0.56	1.00	0.03	1.00	1.00	0.15	0.99	0.82	0.11	0.23	0.001

**Notes.**

*H*_*O*_, Observed Heterozygosity; *H*_*E*_, Expeted Heterozygosity; *p*, significant *p* value for H-W.

**Table 2:** Main genetic pattern estimates from all microsatellites loci of *Inia geoffrensis* individuals from Ariau and Novo Airão localities.

Locality	Genetic index	Values
Ariaú *N* = 7	A_NA_	3.25
	A_R_	3.15
	P_AR_	0.72
	A_GD_	0.58
	*H*_*O*_	0.61
	*H*_*E*_	0.58
	*F*_*IS*_	0.14
Novo Airão *N* = 10	A_NA_	3.92
	A_R_	3.42
	P_AR_	1.00
	A_GD_	0.59
	*H*_*O*_	0.56
	*H*_*E*_	0.58
	*F*_*IS*_	0.08

**Notes.**

N number of individuals A_NA_ average number of alleles per locus A_R_ allelic richness P_AR_ private allelic richness A_GD_ average genetic diversity H_O_ observed heterozygosity H_E_ expected heterozygosity *F*_IS_ inbreeding coefficient

The most likely number of biological groups inferred in the program STRUCTURE was one (ln Pr (*X*|*K* = 1) =  − 447.77 (Fig. S1). Values of K larger than one always had posterior probabilities smaller than that observed for *K* = 1.

The analyses of *F*_*ST*_ revealed the absence of population structuring (*F*_*ST*_ = 0.02429, *P* = 0.66), as noted in STRUCTURE analyses, with greater variation found within groups (87.02%) than between groups (2.43%). Low levels of inbreeding coefficients (*F*_*IS*_) were observed in both groups, 0.001 (*P* = 0.510) for the Novo Airão individuals and −0.120 (*P* = 0.933) for the Ariaú individuals.

We found evidence of high contemporary migration between the two aggregations based on BayesAss results. Migration estimates were of over 25% from Ariaú to Novo Airão, with 0.2890 (*p* = 0.0407), and from Novo Airão to Ariaú at over 5% with 0.0648 (*p* = 0.0480).

### Molecular sexing

Prior to the analysis of relatedness between individuals, the specimens were sexed using molecular markers for the ZFX gene located on the X chromosome and the SRY gene on the Y chromosome developed for cetaceans (Richard, McCarry & Wright, 1994; Bérubé & Palsbøll, 1996; Rosel, 2003). Our molecular sexing analyses indicated that all sampled individuals, both from Ariaú and Novo Airão were males.

### Relatedness

**Figure 2:**
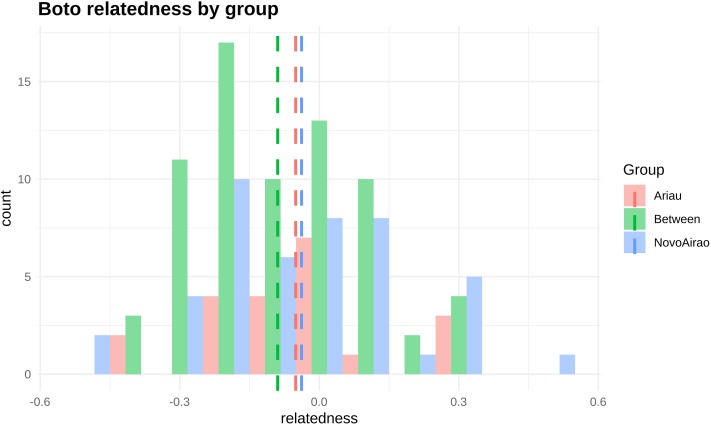
Frequency distribution of relationship values (rQG) based on the results obtained with KINGROUP software. We calculated relatedness (r) values between each pair of individuals from Novo Airão (blue), Ariaú (red) and between individuals of the two aggregations (green). Dashed vertical lines are group means.

KINGROUP analysis showed low mean *r* values (relationship coefficients), both within and between aggregations (−0.03 within Novo Airão, -0.05 within Ariaú and −0.08 between the two locations). Although average values are negative, we can find a certain degree of relatedness of some individuals, both within and between aggregations (Fig. 2). Various coefficient values indicated first, second as well as third order level relationships between individuals. However, after analyzing the *r* values obtained for the pairs of individuals using both softwares, only 23% of comparisons, 34 pairs, had any kind of relationship confirmed. Thus, based on the described criteria to classify relationships, six were considered third order relationships (CO) and seven second order relationships (HS); 21 pairs showed disagreement in relationships inferred in the two programs, and 102 pairs were unrelated (Table S1).

## Discussion

Currently there are seven floating houses provisioning Amazonian river dolphins in the lower Negro River near the city of Manaus. Little is known about the animals that frequent these sites. This was the first study that involved the use of molecular markers to understand the genetic structure and relationship of two of the seven aggregations, and to genetically characterize botos from the Negro River.

### Genetic diversity and population structure

Mean gene diversity (0.58 ± 0.32), *H*_*O*_ (0.58 ±0.20) and *H*_*E*_ (0.59 ± 0.16) of the two aggregations of *Inia geoffrensis* reported here are low, however, they are similar to other populations that have been analyzed in two geographically close areas of the central Amazon, which showed average *H*_*O*_ values of 0.54 ± 0.30 and 0.57 ± 0.36 for Mamirauá Reserve and Tefé lake, respectively (Hollatz et al., 2011). For other *Inia* species heterozygosities are even lower, Gravena (2013) reported *H*_*E*_ = 0.36 for *Inia boliviensis*. Genetic diversity has been treated by the IUCN as a priority parameter for measuring the risk of extinction of a species, since it represents the substratum on which natural selection acts to promote the adaptation of a species to changing environments. The loss of genetic diversity can lead to loss of reproductive fitness and decrease in the evolutionary potential of a given species (Frankham, Ballou & Briscoe, 2010), so the monitoring of this parameter is crucial to direct actions for its conservation. As of 2018, the conservation status of *Inia geoffrensis* has been reassigned to “endangered” by the Brazilian Ministry of the Environment, but is listed as Data Deficient on the IUCN Red List (IUCN Red List of threatened species, 2017). Heterozygosity levels are higher (*H*_*O*_ = 0.53 and *H*_*E*_ = 0.54, Cunha & Watts, 2007; *H*_*O*_ = 0.52 and *H*_*E*_ = 0.59, Lima et al., 2017) for the Data Deficient *Sotalia fluviatilis*, the other Amazonian freshwater dolphin species. On the other hand, the franciscana dolphin (*Pontoporia blainvillei*) is classified as one of the most endangered species of small cetaceans in the southwestern Atlantic Ocean (Secchi, 2010) being listed as Vulnerable on the IUCN Red List, but showing a much higher levels of heterozygosity (*H*_*O*_ = 0.82 and *H*_*E*_ = 0.80, in Costa-Urrutia et al. (2012); *H*_*O*_ = 0.70 and *H*_*E*_ = 0.78, in Gariboldi et al. (2016) than any of the *Inia* species. If low genetic variability is used as a proxy for vulnerability, then *Inia* species should be considered vulnerable, as all the above mentioned species have higher genetic variability.

The results of *F*_*ST*_ values and the STRUCTURE analyses revealed an absence of population structure between the two aggregations sampled in the Negro River. This implies that gene flow between the groups is adequate ensures allelic mixing, as noted with the *Nm* values inferred in this study, despite the approximately 90 km separating both localities. Although botos show high levels of site fidelity (Martin & da Silva, 2004), these values suggest a significant number of alleles being transferred between groups. This may mean that the aggregations are a random subsample of a larger group within the lower Negro River.

Non-significant *F*_*IS*_ values suggests low inbreeding rates in the lower Negro River population. These rates were similar to those observed in the Franciscana dolphins (0.027 and 0.009) (Costa-Urrutia et al., 2012) and higher than the one found by Hollatz et al. (2011), suggesting that reproduction is not restricted to only nearby mates, but draws upon a large pool of individuals.

### Relatedness

Many species of cetaceans have been studied to investigate relationships within migratory groups, alliances or even short-term associations. Some examples include the humpback whale (*Megaptera novaeangliae*) (Valsecchi et al., 2002; Cypriano-Souza et al., 2010), the sperm whale (*Physeter macrocephalus*) (Lyrholm et al., 1999), the bottlenose dolphin (*Tursiops* sp.) (Parsons et al., 2003; Connor et al., 2011), the killer whale (*Orcinus orca*) (Deecke et al., 2010), and the Atlantic white-sided dolphin (*Lagenorhynchus acutus*) (Mirimin et al., 2011), among others. The botos at both Ariaú River and Novo Airão exhibited a certain level of kinship within and between their respective groups. Relationship was found in 34 pairs of 136 comparisons. Six of them are third order relationship (CO), seven are second order relationships (HS), and 21 pairs obtained disagreement between the results of the two programs. Only one of these 21 comparison have a significant probability of being a first order relationship (FS). Seven of them can be either third (CO) or second order relationship (HS), and 13 pairs can be either unrelated or third order relationship (CO).

Only 1 of 136 comparisons were likely to be first order relationship (FS). These are botos 66 and 70 (Rafinha and Ricardo, respectively), both from the Novo Airão locality, most likely being full siblings according to the pattern of allele sharing.

It is impossible to sample all animals that ever frequented the floating houses, however, all animals that were interacting on collection days were sampled. As has been explained before, animals are recognized by scars, pigmentation patterns and other marks by the keepers, and animals that are known in a particular floating house by a name, are known in another floating house by another name. These animals may disappear for some time but be present in another aggregation (Angélica Nunes, Marcele Valle and W.G., pers. com., 2018). For this reason, and the short collection time in each floating house, it was not possible to find all the botos that interact in the aggregations.

Although a certain level of relationship has been observed, we can infer that these animals do not aggregate only with related individuals, as reported for alliances formed in some population of *Tursiops* (Krützen et al., 2003; Parsons et al., 2003). In other cases, such as with *T. aduncus* in southeastern Australia, males generally associate with other males, and the majority of males within these alliances are unrelated (Möller et al., 2001). Analyses of stranded groups of white-sided dolphins (*Lagenorhynchus acutus*), indicate that group formation may occur regardless of the familiar relationship among individuals (Mirimin et al., 2011). A different pattern of relationship has been reported in the franciscana dolphin. These dolphins form small mixed groups of two to six individuals of different ages for the purposes of feeding in the winter and breeding in the spring and summer (Bordino, Thompson & Iniguez, 1999; Crespo, Harris & Gonzáles, 1998). These groups may be composed of either random or highly related individuals, with the presence of an older matriarch and males and females of different ages (Costa-Urrutia et al., 2012). These results show that botos gather at the investigated sites exclusively to receive food, not because they belong to a family group as previously thought by the keepers and owners of the floating houses.

In a previous behavioral study, four botos from the Novo Airão aggregation were sexed by underwater observation of the genital opening position, and were determined to be males (Barezani, 2005). These four botos, Curumin, Fefa, Dani and Lawrence, were sampled in this study, confirming that all four were males, as were all the other botos sampled. Assuming equal frequency of males and females in the regional pool of botos, the probability of having had sampled only males by chance, or the feeding aggregation have had been formed by chance only by males is 7.63 ×10^−6^. Therefore, it is highly likely that only males form these feeding aggregations. Another indicator that these botos are male is that all the animals that regularly congregate at the floating houses present tooth-rake scars over much of their body, a pattern almost exclusively seen in males of this species (Martin & da Silva, 2006).

As no females were observed in the samples of the Novo Airão and Ariaú River aggregations, these results support the habitat preference reported by Martin & da Silva (2004), since both localities are in the main channel of the Negro River. The same authors described that males prefer larger rivers, while females and females with calves remain in more isolated, calmer and protected sites, such as lakes and flooded forests. In the dry season, both sexes are more restricted to the main channel of rivers or large deep lakes. When the water levels rise, individuals disperse into the flooded areas (Best & da Silva, 1993). Births occur asynchronously but are more common during the low-water season, the calf remains with its mother, which can be pregnant and lactating at the same time, for at least two years (da Silva & Martin, 2014; Martin & da Silva, 2018). Mature females spend most of their lives accompanied by a dependent offspring (da Silva & Martin, 2014), which can explain why they do not frequent the aggregations, but remain in protected areas. Mothers with calves prefer calmer areas than where the floating houses are located, which have high traffic of tourism boats, are extremely agitated and noisy during the interactions, and where sometimes botos can be aggressive towards each other.

Most comparisons between individuals resulted in unrelated or inconclusive relationships due to broad confidence intervals of relatedness categories. Only three pairs of individuals were considered full siblings, thus we present evidence to support the conclusion that aggregations are formed for feeding and not based on previously established familial or social relationships. The difference between the natural aggregations and the aggregations analyzed in this study is that until this moment, only males have been found to frequent these artificial aggregations. This changes the social structure, since aggregations in confluence areas are formed by animals of both sexes and from different ages (Martin, da Silva & Salmon, 2004; da Silva & Martin, 2014). Several other dolphin feeding areas used as tourist attractions are known for *Inia* in the Amazon and Tocantins-Araguaia basins (authors pers. obs., 2018). The effects of this interaction on species’ behavior are still not known. Therefore, health assessment, behavior, bioacoustics, human interaction and photo-identification studies are being carried out in order to better understand the structure of these aggregations and the impacts of human interaction on these animals.

## Conclusion

Of the 136 relationship comparisons, only 34 comparisons showed levels of kinship between botos in and between aggregations. Among them, we observed six pair that are considered cousins, seven pairs that are half siblings, and 21 pairs where assignments disagree between the two programs used. Although we observed few instances of familiar relationships, these results do not contradict the paradigm that aggregations of botos are not familial groups, and that aggregations are formed exclusively to receive food. The effects of this type of interaction between botos and humans are still unknown, and therefore basic biological, ecological and ethological research is necessary to develop a comprehensive understanding of the potential consequences of this newfound interaction. Efforts in this direction have already begun within the scope of the Projeto Botos do Rio Negro.

##  Supplemental Information

10.7717/peerj.6692/supp-1 Table S1 Information used in the classification of relationships Information used in the classification of relationships. Note: “ind1” and “ind2” indicate individuals in the pair tested, where “NA” represents animals from Novo Airão, and “AR” from Ariaú; The relatedness index was obtained using KINGROUP and IDENTIX software; and the final conclusion was based on both analyses into the following relationship classes: parent-offspring (PO), full sibs (FS), half sibs (HS), cousins (CO) and unrelated (UN).

10.7717/peerj.6692/supp-2 Figure S1 Graph representation of Bayesian inference of population structuring inferred in STRUCTURE Graph representation of Bayesian inference of population structuring inferred in STRUCTURE. Highest posterior probability is associated with one biological group of *Inia* (lnPr(*X*|*K* = 1) =  − 447.77).

## Additional Information and Declarations

### Field Study Permissions

The following information was supplied relating to field study approvals (i.e., approving body and any reference numbers): The Instituto Brasileiro do Meio Ambiente (IBAMA) and Instituto Chico Mendes de Conservação da Biodiversidade (ICMBio) approved the field research (approval nos. 11325-1 and 13462-1) .

### Data Availability

The following information was supplied regarding data availability: The matrix of genotypes is available at https://github.com/legalLab/datasets.

## References

[ref-1] Alves LCPS, Andriolo A, Orams MB, Azevedo AF (2011). The growth of ‘botos feeding tourism’, a new tourism industry based on the boto (Amazon river dolphin) *Inia geoffrensis* in the Amazonas State, Brazil. Sitientibus serie Ciências Biológicas.

[ref-2] Alves LCP de S, Andriolo A, Orams MB, Azevedo AF (2013). Resource defense and dominance hierarchy in the boto (*Inia geoffrensis*) during a provisioning program. Acta Ethologica.

[ref-3] Ballance LT, Perrin WF, Würsig B, Thewissen JGM (2009). Cetacean ecology. Encyclopedia of marine mammals.

[ref-4] Barezani CP (2005). Conhecimento local sobre o boto-vermelho, *Inia geoffrensis* (de Blainville, 1817), no Baixo Rio Negro e um estudo de caso de suas interações com humanos. Dissertação (Mestrado).

[ref-5] Belkhir K, Castric V, Bonhomme F (2002). IDENTIX, a software to test for relatedness in a population using permutation methods. Molecular Ecology Notes.

[ref-6] Bérubé M, Palsbøll P (1996). Identification of sex in Cetaceans by multiplexing with three ZFX and ZFY specific primers. Molecular Ecology.

[ref-7] Best RC, da Silva VMF, Perrin WF, Brownell RL (1989a). Status and conservation of *Inia geoffrensis* in the Amazon and Orinoco River Basins. Biology and conservation of the River Dolphins.

[ref-8] Best RC, da Silva VMF, Ridgway SH, Harrison RJ (1989b). Amazon river dolphin, Boto. *Inia geoffrensis* (de Blaiville, 1817). Handbook of marine mammals.

[ref-9] Best RC, da Silva VMF (1993). Inia geoffrensis. Mammalian Species.

[ref-10] Bordino P, Thompson G, Iniguez M (1999). Ecology and behavior of the franciscana dolphin *Pontoporia blainvillei* in Bahia Anegada, Argentina. The Journal of Catacean Research and Management.

[ref-11] Braulik GT, Wilson D, Mittermeier RA (2014). Family Platanistidae (South Asian River Dolphin). Handbook of the mammals of the world.

[ref-12] Caldwell M, Gaines MS, Hughes CR (2002). Eight polymorphic microsatellite loci for bottlenose dolphin and other cetacean species. Molecular Ecology Notes.

[ref-13] Cameron EZ, Setsaas TH, Linklater WL (2009). Social bonds between unrelated females increase reproductive success in feral horses. Proceedings of the National Academy of Sciences of the United States of America.

[ref-14] Connor RC, Watson-Capps JJ, Sherwin WB, Kru M, Krützen M (2011). A new level of complexity in the male alliance networks of Indian Ocean bottlenose dolphins (*Tursiops* sp.). Biology Letters.

[ref-15] Costa-Urrutia P, Abud C, Secchi ER, Lessa EP (2012). Population genetic structure and social kin associations of franciscana folphin, *Pontoporia blainvillei*. Journal of Heredity.

[ref-16] Crespo EA, Harris G, Gonzáles R (1998). Group size and distributional range of the franciscana *Pontoporia blainvillei*. Marine Mammal Science.

[ref-17] Cunha HA, Azevedo AF, Lailson-Brito Jr J (2010). A new skin biopsy system for use with small cetaceans. Latin American Journal of Aquatic Mammals.

[ref-18] Cunha HA, Watts PC (2007). Twelve microsatellite loci for marine and riverine tucuxi dolphins (*Sotalia guianensis* and *Sotalia fluviatilis*). Molecular Ecology Notes.

[ref-19] Cypriano-Souza AL, Fernández GP, Lima-Rosa CAV, Engel MH, Bonatto SL (2010). Microsatellite genetic characterization of the humpback whale (*Megaptera novaeangliae*) breeding ground off Brazil (Breeding Stock A). Journal of Heredity.

[ref-20] da Silva VMF, Martin AR, Trujillo F, Crespo EA, Van Damme PA, Usma JS (2010). Status, threats, conservation initiatives and possible solutions for *Inia geoffrensis* and *Sotalia fluviatilis* in Brazil. The action plan for South American River Dolphins 2010–2020.

[ref-21] da Silva VMF, Martin AR, Wilson DE, Mittermeier RA (2014). Family Iniidae (Amazon River Dolphins). Handbook of the mammals of the world.

[ref-22] Deecke VB, Barrett-Lennard LG, Spong P, Ford JKB (2010). The structure of stereotyped calls reflects kinship and social affiliation in resident killer whales (*Orcinus orca*). Naturwissenschaften.

[ref-23] Excoffier L, Lischer HEL (2010). Arlequin suite ver 3.5: a new series of programs to perform population genetics analyses under Linux and Windows. Molecular Ecology Resources.

[ref-24] Excoffier L, Smouse PE, Quattro JM (1992). Analysis of molecular variance inferred from metric distances among DNA haplotypes: application to human mitochondrial DNA restriction data. Genetics.

[ref-25] Frankham R, Ballou JD, Briscoe DA (2010). Introduction to conservation genetics.

[ref-26] Gaggiotti OE, Lange O, Rassmann K, Gliddon C (1999). A comparison of two indirect methods for estimating average levels of gene flow using microsatellite data. Molecular Ecology.

[ref-27] Gariboldi MC, Túnez JI, Failla M, Hevia M, Panebianco MV, Paso Viola MN, Vitullo AD, Cappozzo HL (2016). Patterns of population structure at microsatellite and mitochondrial DNA markers in the franciscana dolphin (*Pontoporia blainvillei*). Ecology and Evolution.

[ref-28] Gompper ME (1997). Population ecology of the white-nosed coati (*Nasua narica*) on Barro Colorado Island, Panama. Journal of Zoology.

[ref-29] Gompper ME, Gittleman JL, Wayne RK (1998). Dispersal, philopatry, and genetic relatedness in a social carnivore: comparing males and females. Molecular Ecology.

[ref-30] Gravena W (2013). O boto vermelho (*Inia* spp.) nos rios Madeira, Mamoré e Guaporé: distribuição, evolução e estrutura populacional. Tese de doutorado. Programa de Pós-Graduação em Genética, Conservação e Biologia Evolutiva.

[ref-31] Gravena W, Hrbek T, da Silva VMF, Astolfi-Filho S, Farias IP (2009). Microsatellite loci for population and parentage analysis in the Amazon River dolphin (*Inia geoffrensis* de Blainville, 1817). Molecular Ecology Resources.

[ref-32] Heinsohn R, Packer C (1995). Complex cooperative strategies in group-territorial African lions. Science.

[ref-33] Heithaus MR, Dill LM, Perrin WF, Würsig B, Thewissen JGM (2009). Feeding strategies and tactics. Encyclopedia of marine mammals.

[ref-34] Herman LM, Herman LM (1980). Cetacean behaviour: mechanisms and functions.

[ref-35] Hollatz C, Vilaça ST, Redondo RAF, Marmontel M, Baker CS, Santos FR (2011). The Amazon River system as an ecological barrier driving genetic differentiation of the pink dolphin (*Inia geoffrensis*). Biological Journal of the Linnean Society.

[ref-36] Hrbek T, da Silva VMF, Dutra N, Gravena W, Martin AR, Farias IP (2014). A new species of river dolphin from Brazil or: how little do we know our biodiversity. PLOS ONE.

[ref-37] IUCN Red List of threatened species (2017). Version 2017-3. http://www.iucnredlist.org.

[ref-38] Kalinowski ST (2005). Hp-Rare 1.0: a computer program for performing rarefaction on measures of allelic richness. Molecular Ecology Notes.

[ref-39] Kappeler PM, Van Schaik CP (2002). Evolution of primate social systems. International Journal of Primatology.

[ref-40] Konovalov DA, Manning C, Henshaw MT (2004). KINGROUP: a program for pedigree relationship reconstruction and kin group assignments using genetic markers. Molecular Ecology Notes.

[ref-41] Krützen M, Sherwin WB, Connor RC, Barré LM, Van de Casteele T, Mann J, Brooks R (2003). Contrasting relatedness patterns in bottlenose dolphins (*Tursiops* sp.) with different alliance strategies. Proceedings of the Royal Society of London Series B: Biological Sciences.

[ref-42] Krützen M, Valsecchi E, Connor RC, Sherwin WB (2001). Characterization of microsatellite loci in *Tursiops aduncus*. Molecular Ecology Notes.

[ref-43] Layne JN, Raymond MAV (1994). Communal nesting of southern flying squirrels in Florida. Journal of Mammalogy.

[ref-44] Leberg PL (2002). Estimating allelic richness: effects of sample size and bottlenecks. Molecular Ecology.

[ref-45] Lima JY, Machado FB, Farro APC, Barbosa LDA, Da Silveira LS, Medina-Acosta E (2017). Population genetic structure of Guiana dolphin (*Sotalia guianensis*) from the southwestern Atlantic coast of Brazil. PLOS ONE.

[ref-46] Lunardi DG, Ferreira RG (2014). Fission-fusion dynamics of Guiana dolphin (*Sotalia guianensis*) groups at Pipa Bay, Rio Grande do Norte, Brazil. Marine Mammal Science.

[ref-47] Lynch M, Ritland K (1999). Estimation of pairwise relatedness with molecular markers. Genetics.

[ref-48] Lyrholm T, Leimar O, Johhanneson B, Gyllensten UB (1999). Sex-biased dispersal in sperm whales: contrasting mitochondrial and nuclear genetic structure of global populations. Proceedings of the Royal Society of London Series B: Biological Sciences.

[ref-49] Martin AR, da Silva VMF (2004). River dolphin and flooded forest: seasonal habitat use and sexual segregation of botos (*Inia geoffrensis*) in an extreme cetacean environment. Journal of Zoology.

[ref-50] Martin AR, da Silva VMF (2006). Sexual dimorphism and body scarring in the boto (Amazon River dolphin) *Inia geoffrensis*. Marine Mammal Science.

[ref-51] Martin AR, da Silva VMF (2018). Reproductive parameters of the Amazon river dolphin or boto, *Inia geoffrensis* (Cetacea: Iniidae); an evolutionary outlier bucks no trends. Biological Journal of the Linnean Society.

[ref-52] Martin AR, da Silva VMF, Salmon DL (2004). Riverine habitat preferences of botos (*Inia geoffrensis*) and tucuxis (*Sotalia fluviatilis*) in the Central Amazon. Marine Mammal Science.

[ref-53] McGuire TL, Henningsen T (2007). Movement patterns and site fidelity of river dolphins *Inia geoffrensis* and *Sotalia fluviatilis* in the Peruvian Amazon as determined by photo-identification. Aquatic Mammals.

[ref-54] Mirimin L, Banguera-Hinestroza E, Dillane E, Hoelzel AR, Cross TF, Rogan E (2011). Insights into genetic diversity, parentage, and group composition of Atlantic white-sided dolphins (*Lagenorhynchus acutus*) off the west of Ireland based on nuclear and mitochondrial genetic markers. The Journal of Heredity.

[ref-55] Möller LM, Beheregaray LB, Harcourt RG, Krutzen M (2001). Alliance membership and kinship in wild male bottlenose dolphins (*Tursiops aduncus*) of southeastern Australia. Proceedings of the Royal Society B: Biological Sciences.

[ref-56] Neigel JE (2002). Is Fst obsolete?. Conservation Genetics.

[ref-57] Norris KS, Dohl TP (1980). The behaviour of the Hawaiian spinner porpoise, *Stenella longirostris*. Fisheries Bulletin.

[ref-58] Paetkau D, Calvert W, Stirling I, Strobeck C (1995). Microsatellite analysis of population structure in Canadian polar bears. Molecular Ecology.

[ref-59] Parsons KM, Durban JW, Claridge DE, Balcomb KC, Noble LR, Thompson PM (2003). Kinship as a basis for alliance formation between male bottlenose dolphins, *Tursiops truncatus*, in the Bahamas. Animal Behaviour.

[ref-60] Pericchi LR, Dey DK, Rao CR (2005). Model selection and hypothesis testing based on objective probabilities and Bayes Factors. Bayesian thinking: modeling and computation.

[ref-61] Pritchard JK, Stephens MJ, Donnelly P (2000). Inference of population structure using multilocus genotype data. Genetics.

[ref-62] Pusey AE, Packer C, Smuts BB, Cheney DL, Seyfarth RM, Wrangham RW, Struhsaker TT (1987). Dispersal and philopatry. Primate societies.

[ref-63] Queller DC, Goodnight KF (1989). Estimating relatedness using genetic markers. Evolution.

[ref-64] Richard KR, McCarry SW, Wright JM (1994). DNA sequence from the SRY gene of the sperm whale (*Physeter macrocephalus*) for use in molecular sexing. Canadian Journal of Zoology.

[ref-65] Romagnoli FC (2010). Interpretação ambiental e envolvimento comunitário: Ecoturismo como ferramenta para a conservação do boto-vermelho, *Inia geoffrensis*. [s.l.]. Master’s dissertation in ecology.

[ref-66] Rosel PE (2003). PCR-based sex determination in Odontocete cetaceans. Conservation Genetics.

[ref-67] Rosel PE, Forgetta V, Dewar K (2005). Isolation and characterization of twelve polymorphic microsatellite markers in bottlenose dolphins (*Tursiops truncatus*). Molecular Ecology Notes.

[ref-68] Schuelke M (2000). An economic method for the fluorescent labeling of PCR fragments. Nature Biotechnology.

[ref-69] Secchi ER, Ruiz-García M, Shostell JM (2010). Review on the threats and conservation status of franciscana, *Pontoporia blainvillei* (Cetacea, Pontoporiidae). Biology, evolution and conservation of river dolphins within South America and Asia.

[ref-70] Silk JB, Beehner JC, Bergman TJ, Crockford C, Engh AL, Moscovice LR, Wittig RM, Seyfarth RM, Cheney DL (2009). The benefits of social capital: close social bonds among female baboons enhance offspring survival. Proceedings of the Royal Society B: Biological Sciences.

[ref-71] Slatkin M (1995). Hitchhiking and associative overdominance at a microsatellite locus. Molecular Biology and Evolution.

[ref-72] Smith BD, Wilson D, Mittermeier RA (2014). Family Lipotidae (Baiji). Handbook of the mammals of the world.

[ref-73] Suchard MA, Weiss RE, Sinsheimer JS (2001). Bayesian selection of continuous- time Markov Chain evolutionary models. Molecular Biology and Evolution.

[ref-74] Thouless CR (1995). Long distance movements of elephants in northern Kenya. African Journal of Ecology.

[ref-75] Valsecchi E, Hale P, Corkeron P, Amos W (2002). Social structure in migrating humpback whales (*Megaptera novaeangliae*). Molecular Ecology.

[ref-76] Van Oosterhout C, Hutchinson WF, Wills DPM, Shipley P (2004). Micro-Checker: software for identifying and correcting genotyping errors in microsatellite data. Molecular Ecology Notes.

[ref-77] Weir BS (1996). Genetic data analysis II: methods for discrete population genetic data.

[ref-78] Weir BS, Cockerham CC (1984). Estimating F-statistics for the analysis of population structure. Evolution.

[ref-79] Wilson GA, Rannala B (2003). Bayesian inference of recent migration rates using multilocus genotypes. Genetics.

[ref-80] Wright S (1969). Evolution and the genetics of populations: the theory of gene frequencies.

