# Supplementary figures and images for: Boto (Inia geoffrensis—Cetacea: Iniidae) aggregations in two provisioning sites in the lower Negro River—Amazonas, Brazil: are they related?
Source: PeerJ. 2019 Apr 17;7:e6692. doi: 10.7717/peerj.6692 (PMC6475133; doi:10.7717/peerj.6692)

L(K) (mean  $\pm$  SD)

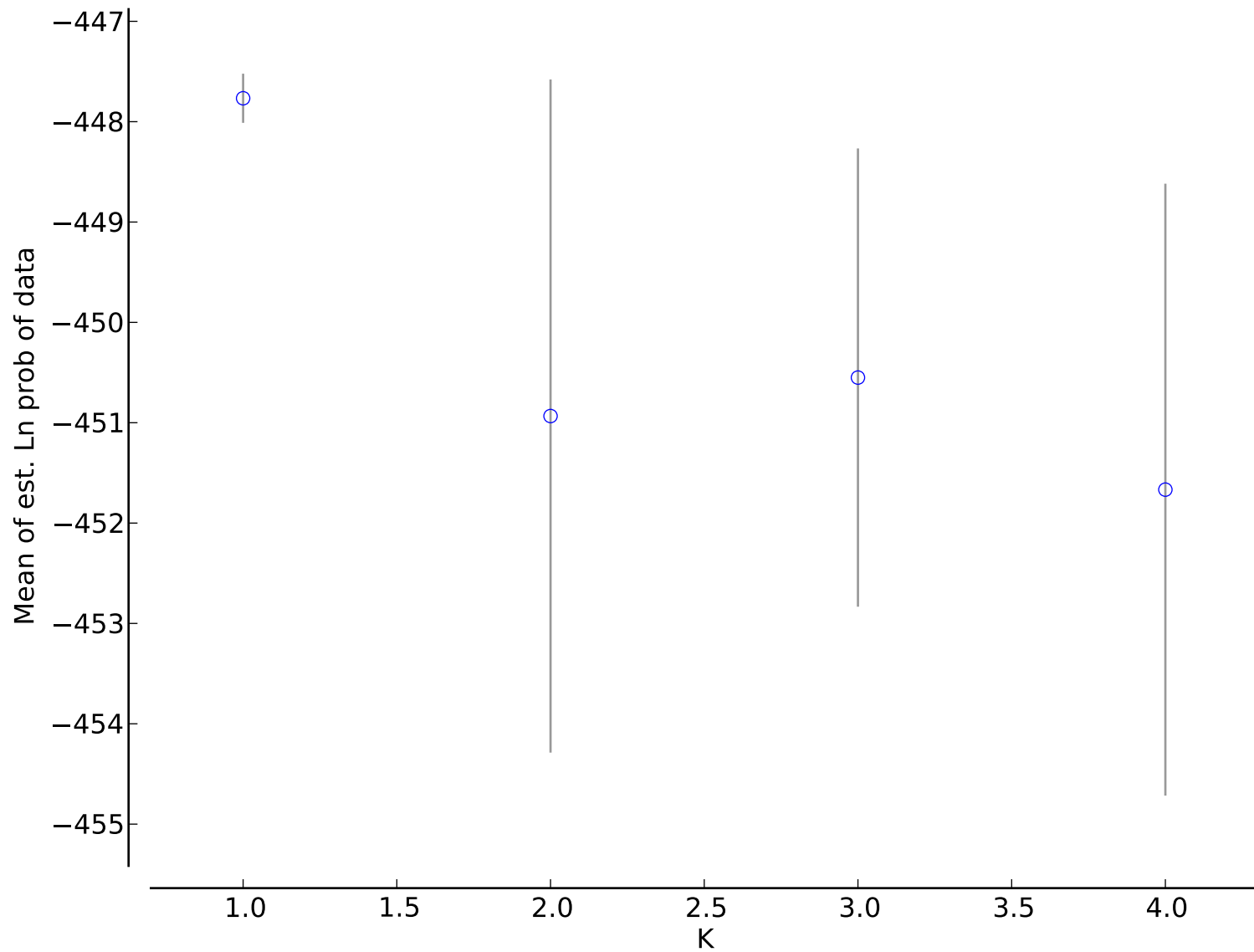

Supplement: Figure S1 — Graph representation of Bayesian inference of population structuring inferred in STRUCTURE. Highest posterior probability is associated with one biological group of Inia (lnPr(X|K = 1) = − 447.77). [file peerj-07-6692-s002.pdf]
